# Feasibility of Hyperspectral Imaging and Machine Learning for Rapid Prescreening of Aflatoxin B_1_ in Maize Kernels

**DOI:** 10.3390/toxins18070308

**Published:** 2026-07-15

**Authors:** Yongping Jiang, Bowen Tai, Yufan Yang, Xinyue Zhang, Jing Jin, Fuguo Xing

**Affiliations:** 1 Institute of Food Science and Technology, Chinese Academy of Agricultural Sciences, Beijing 100193, China; 2 College of Light Industry and Food, Zhongkai University of Agriculture and Engineering, Guangzhou 510000, China

**Keywords:** hyperspectral imaging, aflatoxin B1, spectral preprocessing, contamination grading, maize kernels

## Abstract

Aflatoxin B_1_ (AFB_1_) contamination in maize poses serious risks to food and feed safety; however, conventional laboratory-based assays are often constrained by time and throughput for large-scale screening. This study proposes a hyperspectral imaging (HSI) workflow for rapid, non-destructive prediction of AFB_1_. A total of 236 hyperspectral images were acquired in the 400–1000 nm range (256 bands) from maize samples covering a broad gradient of AFB_1_ contamination, and spectral features were extracted from regions of interest (ROI) for model development. The results demonstrate that appropriate spectral preprocessing and wavelength selection play a critical role in improving model robustness, with the SNV–CARS–KNN model achieving the best prediction performance (test R^2^ = 0.9341, RMSE = 4.8938). Based on the predicted AFB_1_ values, contamination grading was further explored to enable rapid screening and risk management in agricultural applications. Aflatoxin B_1_ (AFB_1_) contamination in maize poses substantial risks to food and feed safety, creating a need for rapid screening tools that can support high-throughput prescreening. In this study, hyperspectral imaging (HSI) was explored as a non-destructive approach for the preliminary assessment of AFB_1_ contamination in maize kernels. A total of 236 hyperspectral images were collected in the 400–1000 nm range, and region-of-interest spectra were extracted for model development. Spectral preprocessing and wavelength selection strategies were compared in combination with several conventional regression models to examine their influence on predictive performance using the current dataset. Among the tested combinations, the SNV–CARS–KNN model showed the most favorable performance on the held-out test set (R^2^ = 0.9341; RMSE = 4.8938). In addition, a grade-based classification derived from predicted values was explored as an application-oriented extension for rapid risk sorting. However, the study was conducted on artificially contaminated samples and relied on rapid-test-derived reference values, so the findings should be interpreted as a proof of concept rather than a fully validated quantitative method. Overall, the results support the potential of HSI for rapid prescreening of AFB_1_ in maize and provide a basis for further validation using more rigorous reference analysis and independent sample sets.

## 1. Introduction

Maize is one of the major cereal commodities vulnerable to aflatoxin contamination, among which aflatoxin B1 (AFB_1_) is generally regarded as the most toxic and hazardous analogue [1]. AFB_1_ contamination in maize is of particular concern because it may occur during both pre-harvest and post-harvest stages, persist during storage and handling, and subsequently enter the food and feed chain [2]. In addition to its toxicological significance and association with serious health risks, AFB_1_ contamination can also lead to substantial economic losses through product rejection, quality downgrading, and trade restrictions [3]. Therefore, the development of rapid screening approaches for maize is important for timely risk identification and lot segregation in practical grain management.

At present, chromatographic methods such as HPLC and LC–MS/MS remain the principal approaches for accurate aflatoxin determination, while immunoassay-based methods are widely used for rapid testing. However, these methods generally rely on sampling, extraction, reagent consumption, and laboratory- or kit-based analytical workflows, which may limit their suitability for rapid, high-throughput, and non-destructive prescreening at the kernel or batch level. In this context, hyperspectral imaging (HSI), which combines spectral information with spatial characteristics, has attracted increasing attention as a potential tool for food safety inspection [4,5,6]. Previous studies have shown that HSI can capture chemical- and structure-related variations in agricultural materials and has been explored for the detection of fungal infection and mycotoxin contamination in grains, including maize [7].

Despite this potential, reliable interpretation of hyperspectral data remains challenging because maize kernel spectra are easily affected by surface morphology, scattering effects, baseline drift, and instrumental noise [8]. Spectral preprocessing is therefore commonly applied to reduce non-chemical variation before model construction, while wavelength-selection strategies are often used to alleviate collinearity and redundancy in full-band data. Methods such as standard normal variate (SNV), multiplicative scatter correction (MSC), competitive adaptive reweighted sampling (CARS) [9], and uninformative variable elimination (UVE) have been widely employed in chemometric analysis of spectral datasets. Even so, the performance of a given workflow can still vary considerably depending on sample characteristics, spectral range, model structure, and reference-label quality. For this reason, comparative evaluation of preprocessing, variable selection, and modeling strategies remains necessary when developing application-oriented HSI workflows [10,11].

Against this background, the present study explored the feasibility of using HSI for rapid, non-destructive prescreening of AFB_1_ contamination in maize under controlled experimental conditions [12,13,14]. Hyperspectral images covering a broad contamination gradient were acquired, and the effects of spectral preprocessing, wavelength selection, and conventional regression models were comparatively examined at the image level [15,16]. In addition, predicted AFB_1_ values were further converted into grade-based categories to preliminarily assess the potential of the workflow for risk-oriented sorting [17]. Rather than establishing a fully validated quantitative method, this work was designed as a proof-of-concept study intended to evaluate whether an HSI-based workflow could provide useful screening information for AFB_1_-contaminated maize and offer a basis for subsequent validation with more rigorous reference analysis and independent sample sets.

## 2. Results

### 2.1. Effects of Spectral Preprocessing on Full-Band PLSR Performance

**Figure 1:**
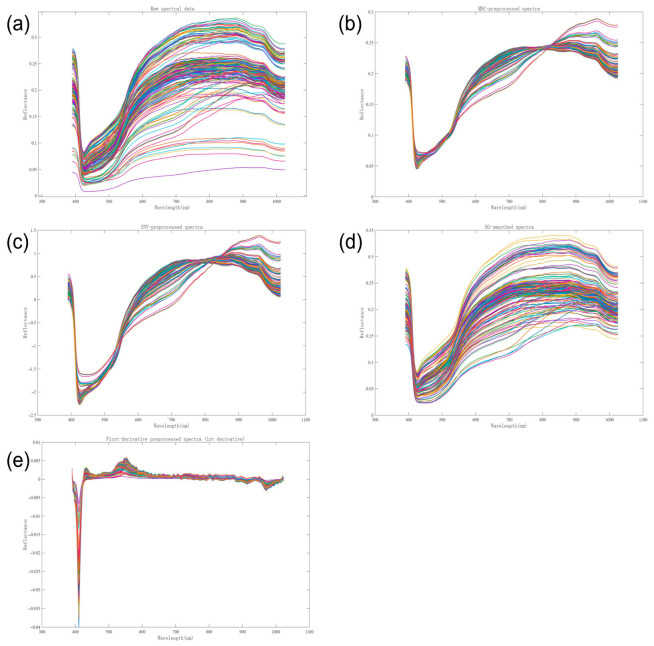
Spectral profiles of maize kernels under different preprocessing methods: (**a**) raw spectra, (**b**) MSC-preprocessed spectra, (**c**) SNV-preprocessed spectra, (**d**) SG-smoothed spectra, and (**e**) first-derivative spectra.

To evaluate the influence of various spectral preprocessing techniques on AFB_1_ prediction in maize kernels, Partial Least Squares Regression (PLSR) models were constructed using full-band hyperspectral data (256 bands) under five preprocessing scenarios: raw spectra, multiplicative scatter correction (MSC), standard normal variate (SNV), Savitzky–Golay (SG) smoothing, and first-derivative transformation. The results of these preprocessing steps are presented in Figure 1.

The raw spectra (Figure 1a) exhibited pronounced baseline offsets and intensity variations among samples, indicating that scattering effects and sample heterogeneity introduced non-chemical spectral variations. Such variations are commonly observed in hyperspectral measurements of agricultural materials and may hinder accurate quantitative modeling if not corrected through spectral preprocessing [18,19]. After applying scatter-correction techniques, the spectral profiles became more consistent. Specifically, MSC effectively reduced inter-sample variation by correcting for multiplicative scattering effects (Figure 1b), while SNV further standardized spectral magnitude and baseline, enhancing the alignment across samples (Figure 1c). SG smoothing primarily suppressed high-frequency noise but did not explicitly correct for scattering or baseline shifts (Figure 1d), whereas first-derivative transformation attenuated baseline contributions and enhanced local spectral changes but amplified noise (Figure 1e), potentially degrading the overall signal-to-noise ratio [20,21,22].

**Figure 2:**
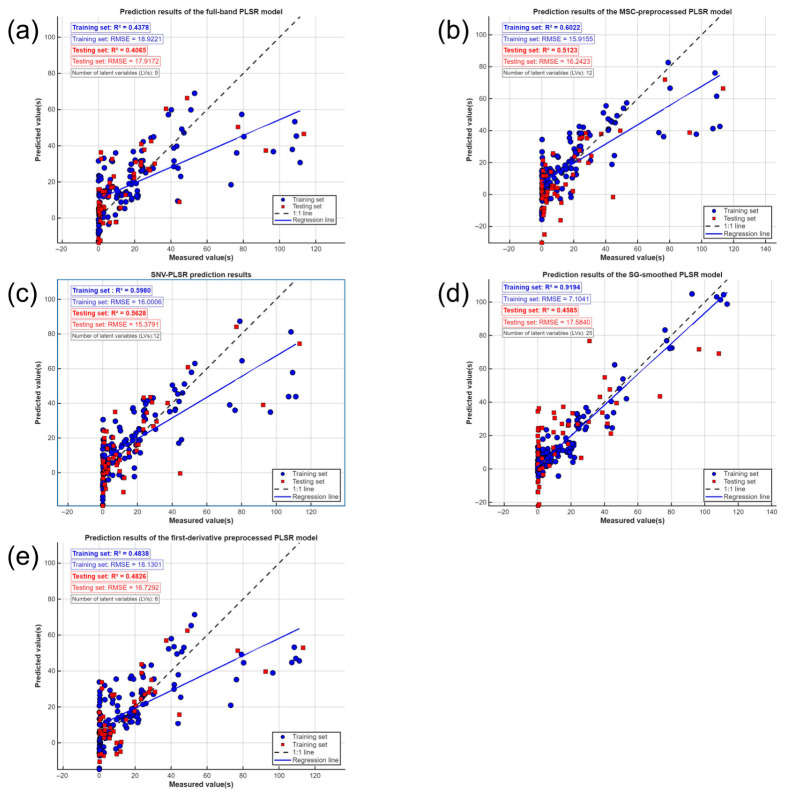
Prediction performance of full-band PLSR models under different preprocessing methods: (**a**) raw spectra; (**b**) MSC-preprocessed spectra; (**c**) SNV-preprocessed spectra; (**d**) SG-smoothed spectra; (**e**) first-derivative spectra.

The predictive performance of the PLSR models built on different spectral preprocessing strategies is summarized in Figure 2. The model based on raw spectra demonstrated limited performance on the test set (R^2^ = 0.4065, RMSE = 17.9172; LVs = 9; Figure 2a). MSC preprocessing moderately improved generalization (R^2^ = 0.5123, RMSE = 16.2423; LVs = 12; Figure 2b), while SNV preprocessing provided the best overall performance on the test set (R^2^ = 0.5628, RMSE = 15.3791; LVs = 12; Figure 2c). These results suggest that SNV not only stabilized spectral intensity but also enhanced the model accuracy.

In contrast, SG smoothing, although producing excellent fitting on the training set (training R^2^ = 0.9194, RMSE = 7.1041), exhibited substantial overfitting, as evidenced by its poor performance on the test set (test R^2^ = 0.4585, RMSE = 17.5840) and the high number of latent variables (LVs = 25; Figure 2d). The first-derivative transformation (Figure 2e) provided moderate improvement over raw spectra (R^2^ = 0.4826, RMSE = 16.7292; LVs = 6), but its overall predictive power was still lower than that of the SNV-preprocessed model.

Given these results, SNV preprocessing showed the most favorable test-set performance under the current train–test split and was therefore used for subsequent wavelength selection and regression modeling. However, this comparison was based on a single dataset partition, and the relative advantage of SNV should be further verified using repeated stratified resampling or external validation.

### 2.2. Wavelength Selection Using CARS and UVE Under SNV-PLSR

**Figure 3:**
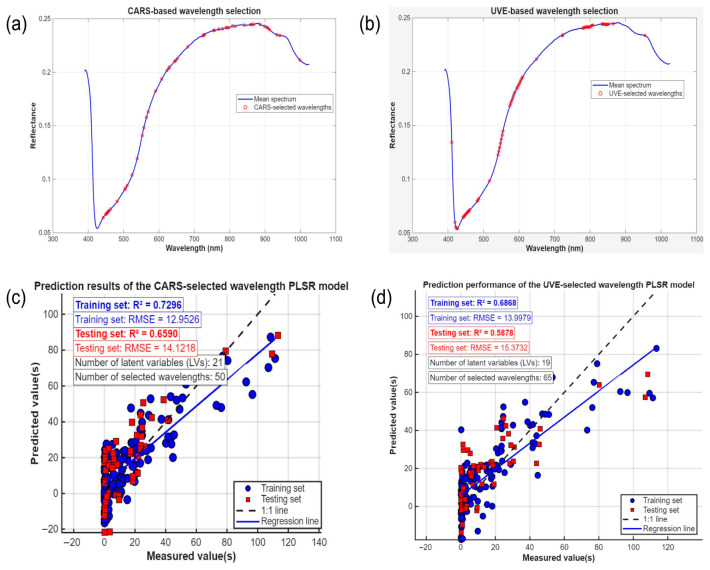
Wavelength selection and prediction performance of SNV-preprocessed partial least squares regression (PLSR) models based on different variable selection methods: (**a**) CARS-based wavelength selection, (**b**) UVE-based wavelength selection, (**c**) prediction performance of the PLSR model using CARS-selected wavelengths, and (**d**) prediction performance of the PLSR model using UVE-selected wavelengths.

After selecting SNV as the optimal preprocessing method, CARS and UVE wavelength selection were applied to reduce spectral dimensionality and improve AFB_1_ prediction in maize kernels. The selected wavelengths are illustrated on the mean spectrum in Figure 3a,b. CARS retained 50 wavelengths (Figure 3a) from the original 256 bands, providing a more compact set of variables compared to UVE, which retained 65 wavelengths (Figure 3b), indicating a stronger dimensionality reduction achieved by CARS. The retained wavelengths are indicated by the red circles in Figure 3a,b, marking key bands identified through each wavelength selection method. These selected wavelengths were mainly distributed in spectral regions related to changes in kernel color, moisture, and major biochemical constituents. Wavelengths in the visible region may reflect surface discoloration, pigment degradation, and fungal growth on maize kernels, whereas those in the near-infrared region are generally associated with overtone and combination absorptions of O-H, C-H, and N-H bonds, which are related to water, carbohydrates, proteins, and lipids. Therefore, the selected wavelengths may represent both the spectral response of contaminated kernels and the biochemical changes induced by Aspergillus flavus growth and AFB_1_ accumulation. It should be noted that AFB_1_ has relatively weak and overlapping absorption features in the visible-near-infrared range; therefore, the selected wavelengths should be interpreted mainly as contamination-related characteristic bands of the maize matrix rather than as unique absorption peaks of AFB_1_. Following the wavelength selection process, we evaluated the performance of SNV-preprocessed PLSR models using both full-band spectra and the selected wavelengths (CARS and UVE). The corresponding results are shown in Figure 3c,d and summarized in Table 1.

Table 1 presents the prediction performance of the models, comparing the use of full-band spectra and the wavelengths selected by CARS and UVE under the SNV preprocessing method. For the full-band SNV-PLSR model, the testing R^2^ was 0.5628, with an RMSE of 15.3791. By contrast, CARS-based wavelength selection improved model generalization, achieving a test-set R^2^ of 0.6590 and RMSE of 14.1218 with 21 latent variables. This demonstrates that CARS not only reduced the number of input wavelengths (from 256 to 50) but also improved predictive accuracy, making it the most effective wavelength selection method. In comparison, UVE-based wavelength selection resulted in a slight improvement in the training set (training R^2^ = 0.6868, RMSE = 13.9979), but the gain on the test set was modest, with a testing R^2^ of 0.5878 and RMSE of 15.3732, using 19 latent variables. Although UVE successfully reduced dimensionality and enhanced the model fit for the training set, its performance on the test set did not surpass that of the CARS-selected wavelengths.

In the present dataset, CARS showed better performance than UVE under the SNV–PLSR framework, producing a more compact wavelength subset and higher test-set accuracy. Therefore, CARS-selected wavelengths were used as input variables for subsequent regression model comparison. Nevertheless, this result should be interpreted within the current sample set and modeling conditions rather than as evidence that CARS is universally superior for AFB_1_-related HSI analysis.

### 2.3. Comparison of Regression Models Based on CARS-Selected Wavelengths

**Figure 4:**
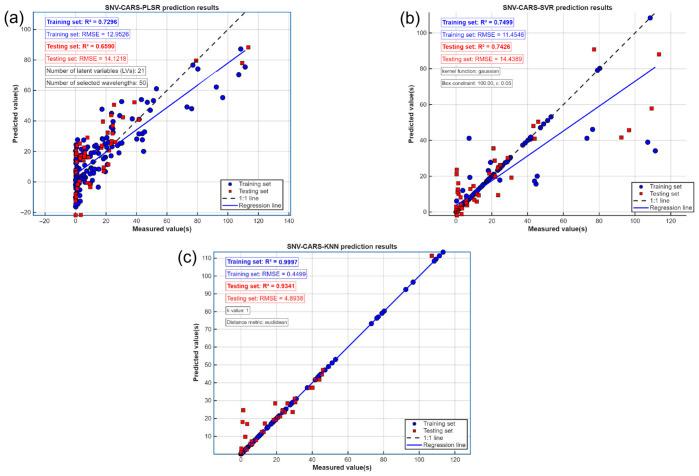
Prediction performance of regression models based on CARS-selected wavelengths using SNV preprocessing: (**a**) partial least squares regression (PLSR), (**b**) support vector regression (SVR), and (**c**) k-nearest neighbors (KNN).

Following the optimal preprocessing with SNV, we performed wavelength selection using the CARS method to reduce dimensionality and enhance model performance for AFB_1_ prediction in maize kernels. Subsequently, three regression models (PLSR, SVR, and KNN) were compared to assess their predictive accuracy based on the CARS-selected wavelengths. The measured–predicted scatter plots for each model are presented in Figure 4a–c, and the corresponding performance metrics are summarized in Table 2. The CARS-PLSR model yielded a training R^2^ of 0.7296 (RMSE = 12.9526) and a testing R^2^ of 0.6590 (RMSE = 14.1218) (Figure 4a). The CARS-SVR model improved the testing R^2^ to 0.7426, while the testing RMSE remained at a similar level (14.4389) (Figure 4b). Among the evaluated models, the CARS-KNN model achieved the highest testing accuracy, with a testing R^2^ of 0.9341 and testing RMSE of 4.8938 (Figure 4c). The training performance of KNN was R^2^ = 0.9997 and RMSE = 0.4499, whereas the test-set results remained markedly better than those of PLSR and SVR.

Overall, using the same SNV preprocessing and CARS-selected wavelength input, KNN showed the most favorable test-set performance in the current dataset. However, the KNN model also produced near-perfect fitting on the training set, indicating a substantial risk of overfitting or dataset-specific memorization. Therefore, the KNN results should be interpreted as exploratory rather than as definitive evidence of model superiority.

### 2.4. Application of Predicted AFB_1_ Values for Contamination Grading

To further evaluate the practical potential of the HSI workflow for rapid risk-oriented sorting, contamination grade classification was performed using the four predefined AFB_1_ grade labels. Unlike the continuous regression analysis, this classification task aimed to assign each sample directly into a risk grade rather than to estimate its exact AFB_1_ concentration. The RF, SVM, and KNN classifiers were developed using the SNV-preprocessed CARS-selected spectral features, and their performance on the independent test set is summarized in Table 3.

**Figure 5:**
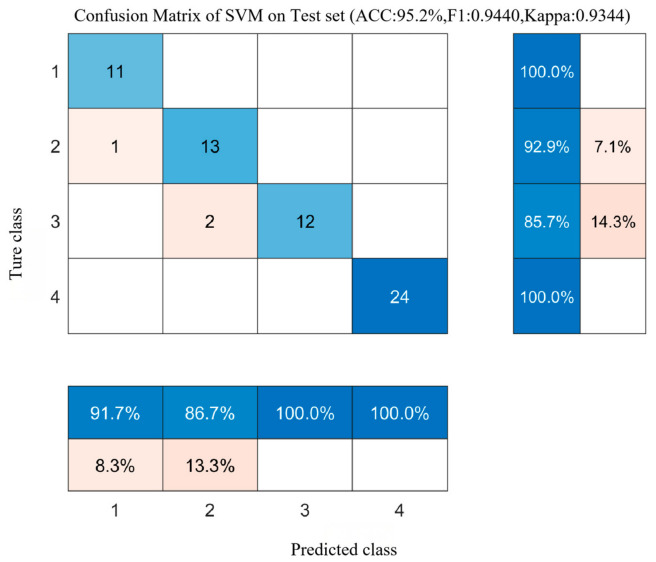
Application-oriented contamination grading based on predicted AFB_1_ values. Confusion matrix of contamination grades (G1–G4) on the independent test set (SVM).

Among the evaluated classification models, SVM achieved the highest overall performance, with an accuracy of 95.24%, a macro-F1 score of 0.9440, and Cohen’s kappa of 0.9344. RF and KNN achieved accuracies of 88.89% and 90.48%, respectively. The confusion matrix of the SVM classifier is shown in Figure 5, and the comparison of Cohen’s kappa values among the three classifiers is presented in Table 3.

These results suggest that HSI-derived spectral features may support preliminary contamination grade classification for rapid risk-oriented screening. However, because classification errors were mainly concentrated near adjacent grade boundaries, samples close to the decision thresholds should still be subjected to confirmatory analysis before regulatory or commercial decisions are made.

## 3. Discussion

This study explored the feasibility of combining hyperspectral imaging with conventional chemometric modeling for rapid prescreening of AFB_1_ contamination in maize kernels under controlled experimental conditions. Within the current dataset, spectral preprocessing and wavelength selection had a noticeable influence on model performance [23,24]. Among the evaluated preprocessing strategies, SNV yielded the most favorable test-set performance in the full-band PLSR comparison, suggesting that reducing scatter-related variation and baseline instability was beneficial for subsequent regression analysis [25]. This observation is consistent with the general role of preprocessing in improving the interpretability and comparability of hyperspectral data prior to model construction [26,27].

Wavelength selection further affected predictive performance under the SNV-PLSR framework. Compared with the full-band model, both CARS and UVE reduced spectral dimensionality, but CARS produced a more compact variable subset and was associated with better test-set performance in the present study. This result suggests that appropriate variable reduction may help alleviate redundancy in full-spectrum data and improve the efficiency of subsequent modeling [28,29,30]. However, the advantage observed here should still be interpreted within the context of the current sample set and modeling conditions, rather than as definitive evidence that one wavelength-selection strategy is universally superior for AFB_1_-related hyperspectral analysis [31]. When different regression models were compared using the CARS-selected wavelengths, the KNN model showed the most favorable test-set performance among the evaluated approaches [32]. However, this result should be interpreted cautiously because the SNV-CARS-KNN model with k = 1 and Euclidean distance obtained a training R^2^ of 0.9997 and a training RMSE of 0.4499, while the testing R^2^ and RMSE were 0.9341 and 4.8938, respectively, indicating a substantial risk of overfitting or dataset-specific memorization rather than stable predictive learning [33]. Therefore, the present findings are more appropriately viewed as an exploratory indication that HSI-derived spectral features may contain information related to AFB_1_ variation in the current sample set, rather than as evidence of a fully established quantitative model with confirmed robustness [34].

The contamination-grading analysis provides an additional application-oriented perspective. By converting predicted AFB_1_ values into G1–G4 categories, the workflow was preliminarily extended from continuous-value prediction to rapid risk-oriented sorting. Most classification errors were concentrated near adjacent grade boundaries, suggesting that the proposed strategy may be more suitable for prescreening and prioritization than for direct compliance judgment in borderline samples [35]. In practical terms, this type of framework may be useful for identifying potentially high-risk samples that should be subjected to confirmatory analysis [36].

Several limitations should be acknowledged. First, the sample set was generated under controlled artificial inoculation and incubation conditions and may not fully represent the heterogeneity of naturally contaminated maize from real storage and supply-chain environments [37]. Second, the reference AFB_1_ values were obtained using an ELISA rapid detection kit rather than chromatographic confirmation. Although ELISA is suitable for rapid screening and practical reference estimation, it may be affected by matrix effects, extraction variability, and kit-specific calibration uncertainty. Therefore, the reported modeling performance should not be interpreted as validation against definitive chromatographic concentrations. Third, model evaluation relied on a single train–test split, and the overall sample size remained limited, so the observed performance may not fully reflect robustness across batches, contamination ranges, or sampling conditions [38]. In addition, the use of ROI-averaged spectra may underrepresent the spatial heterogeneity of localized contamination within individual kernels [39].

Overall, the current results provide preliminary support for the potential of HSI as a rapid and non-destructive tool for AFB_1_ prescreening in maize. Nevertheless, substantial further work is still required before such an approach can be considered analytically robust for broader application. Future studies should prioritize validation with naturally contaminated multi-origin maize, chromatographic reference analysis, repeated model evaluation strategies, and the integration of more spatially resolved information.

## 4. Conclusions

This study provides preliminary evidence that hyperspectral imaging, combined with spectral preprocessing and wavelength selection, may support rapid, non-destructive prescreening of AFB_1_ contamination in maize kernels under controlled experimental conditions. Among the compared modeling strategies, the SNV–CARS–KNN combination showed the most favorable test-set performance within the current dataset. In addition, converting predicted AFB_1_ values into contamination grades suggested potential utility for rapid risk-oriented sorting.

However, the present findings should be interpreted as proof-of-concept results rather than as a fully validated quantitative solution, because the study was based on artificially contaminated samples, ELISA-derived reference estimates, and a single stratified train–test split. Future studies should prioritize validation using chromatographic reference methods such as HPLC or LC–MS/MS, naturally contaminated maize samples from multiple origins, repeated stratified resampling, independent external batches, and threshold-oriented evaluation for regulatory screening scenarios. Incorporating pixel-level or sub-region spectral information may also improve sensitivity to spatially heterogeneous contamination and support future high-throughput sorting applications.

## 5. Materials and Methods

### 5.1. Sample Preparation

A total of 236 maize kernel sample groups were prepared. After artificial inoculation with a toxigenic Aspergillus flavus suspension, the samples were incubated at 21, 28, and 37 °C, respectively, with 78 samples assigned to each temperature condition for 7 consecutive days. Sampling followed a daily monitoring scheme. Hyperspectral images were acquired once per day during the incubation period, and AFB_1_ concentration was measured on the same day to construct a dataset spanning AFB_1_ levels from non-detectable to heavily contaminated.

Reference AFB_1_ values were determined using an aflatoxin B1 ELISA rapid detection kit (Hua’an Maike Biotechnology Co., Ltd., Cat. No. HEM0496, 96 tests/kit, Beijing, China) according to the manufacturer’s instructions. Briefly, maize samples were prepared and extracted following the kit protocol, and the AFB_1_ concentration was calculated based on the kit-specific calibration procedure. Each hyperspectral image was paired with its corresponding ELISA-derived AFB_1_ value, which was used as the reference label for subsequent regression modeling.

Because chromatographic confirmation methods such as HPLC or LC–MS/MS were not applied, the ELISA-derived values were used as screening-oriented reference estimates rather than definitive quantitative determinations. Therefore, the present work should be interpreted as a proof-of-concept study for rapid prescreening rather than as a fully validated quantitative analytical method.

### 5.2. Hyperspectral Imaging System and Image Acquisition

Hyperspectral images of maize kernels were acquired using a GaiaField-V10E hyperspectral imaging system.(Sichuan Dualix Spectral Imaging Technology Co., Ltd., Chengdu, China) The system covered a spectral range of 400–1000 nm, with a spectral resolution of 2.8 nm and a sampling interval of 0.7 nm. The spatial resolution of the acquired images was 1392 × 1400 pixels. Image acquisition was performed inside an HSIA-DB hyperspectral dark box equipped with a tungsten–halogen lamp (rated power: 400 W) to minimize ambient light interference and ensure stable illumination. A push-broom scanning mode was used for hyperspectral imaging. White reference calibration was conducted prior to image acquisition. After acquisition, lens calibration and reflectance calibration were further applied to reduce lens-related imaging errors and to correct for illumination and instrument response variations. The calibrated data were used for subsequent analysis and modeling.

### 5.3. ROI Selection and Spectral Extraction

Hyperspectral images were processed using ENVI 5.6. For each image, the region corresponding to an individual maize kernel was manually delineated as the region of interest (ROI). The contamination level of each sample was then annotated according to its corresponding AFB_1_ measurement, and the labeled ROI was saved for subsequent modeling. Spectral information was extracted from the ROI by calculating the mean spectrum of all pixels within the ROI, which served as the representative spectral feature for each hyperspectral image. In this way, each hyperspectral image was converted into a one-dimensional spectral vector for subsequent preprocessing, wavelength selection, and regression modeling.

### 5.4. Dataset Partitioning and Stratified Sampling

For the regression modeling task, the distribution of reference AFB_1_ concentrations was first examined before dataset partitioning. Because low-concentration samples accounted for a relatively high proportion of the original dataset, directly including all samples in the regression model could have caused the model to overfit the spectral characteristics of low-contamination samples and reduced its predictive ability for high-concentration and over-limit samples. Therefore, samples with abnormal reference values or abnormal spectral responses were first excluded according to the quality-control criteria established during data preprocessing. The remaining samples were then grouped into five concentration intervals according to their reference AFB_1_ values. To reduce the influence of distribution imbalance, the number of samples in the extremely low-AFB_1_ interval was moderately adjusted by random undersampling, while samples in the medium- and high-concentration intervals were retained as far as possible. No synthetic samples were generated during this process. Finally, stratified random sampling was performed within each concentration interval, and the dataset was divided at a ratio of 7:3 into a training set containing 124 samples and a testing set containing 53 samples.

### 5.5. Spectral Preprocessing

To reduce the influence of non-chemical variations and to enhance AFB_1_-related information, spectral preprocessing was evaluated on the ROI-averaged spectra. The preprocessing methods included multiplicative scatter correction (MSC), standard normal variate (SNV), Savitzky–Golay (SG) smoothing, and first-derivative transformation. For a fair comparison, partial least squares regression (PLSR) built on full-band spectra was used as the baseline framework. All preprocessing strategies were benchmarked using the same dataset split and evaluation criteria to ensure comparability. Based on this framework, the effects of different preprocessing methods on model performance were subsequently analyzed.

### 5.6. Feature Wavelength Selection

To reduce high-dimensional redundancy and multicollinearity in full-band spectra and to improve modeling efficiency and robustness, feature wavelength selection was performed on SNV-preprocessed spectra. Two methods—CARS and UVE—were evaluated. CARS performs iterative model fitting under Monte Carlo sampling and selects wavelengths in a competitive manner according to their contributions, where less-informative variables are gradually removed via an exponentially decreasing function. The optimal wavelength subset is determined by the minimum cross-validation error (RMSECV). In this study, CARS selected 50 informative wavelengths from the original 256 bands. UVE introduces random noise variables and assesses the stability of regression coefficients to establish a threshold for identifying uninformative variables, thereby removing wavelengths with low contributions. In this study, UVE selected 65 wavelengths. Subsequent regression modeling was conducted based on each selected feature set to evaluate the impact of wavelength selection strategies on AFB_1_ prediction performance.

### 5.7. Regression Models and Parameter Tuning

Three commonly used regression models were evaluated in this study, including partial least squares regression (PLSR), support vector regression (SVR), and k-nearest neighbors regression (KNN). These models were selected because they represent widely used chemometric and machine learning approaches for spectral data analysis.

For KNN regression, Euclidean distance was used as the distance metric, and the number of nearest neighbors was set to k = 1 in the current exploratory comparison. This setting produced the most favorable test-set performance under the present dataset partition. However, because k = 1 can lead to near-perfect fitting of the training samples, it may increase the risk of overfitting or dataset-specific memorization. Therefore, the KNN regression result was interpreted as exploratory rather than as definitive evidence of model superiority. Future studies should optimize k using cross-validation with k > 1 and compare the optimized KNN model with SVR and PLSR under repeated resampling and external validation.

All models were implemented and evaluated using the same training–test partition to ensure consistent comparison.

### 5.8. Performance Evaluation

Model performance was evaluated using R^2^ and RMSE, reported for both the training and testing sets. R^2^ measures the proportion of variance in the observations explained by the model:

$$R^{2}=1-\frac{\sum_{i=1}^{n}{\left(y_{i}-{\hat{y}}_{i}\right)}^{2}}{\sum_{i=1}^{n}{\left(y_{i}-\bar{y}\right)}^{2}}$$

where $y_{i}$ is the measured value, ${\hat{y}}_{i}$ is the predicted value, $\bar{y}$ is the mean of measured values, and n is the number of samples. Values closer to 1 indicate a better fit. RMSE quantifies the magnitude of prediction errors:

$$\mathrm{RMSE}=\sqrt{\frac{1}{n}\overset{n}{\underset{i=1}{\sum }}{\left(y_{i}-{\hat{y}}_{i}\right)}^{2}}$$

Lower RMSE indicates better predictive performance.

### 5.9. Software and Implementation

ROI delineation and labeling were conducted in ENVI 5.6. Spectral preprocessing, wavelength selection (CARS and UVE), regression modeling (PLSR, SVR, and KNN), and cross-validation were implemented in MATLAB, where model tuning and comparisons were performed on the training set.

### 5.10. Statistical Analysis

All data processing, statistical analysis, and model development were conducted using MATLAB (R2025a). Model performance was evaluated using the coefficient of determination (R^2^) and the root mean square error (RMSE) for both the training and testing sets. To ensure a fair comparison among different preprocessing, wavelength selection, and regression strategies, the same dataset split and evaluation criteria were consistently applied across all models.

It should be noted that the present study used a single stratified training–testing split for exploratory model evaluation. Repeated stratified resampling, nested cross-validation, or independent external validation was not performed in the present proof-of-concept study. Therefore, the reported performance should be interpreted as preliminary evidence obtained using the current dataset partition rather than as a definitive estimate of generalization performance.

## Figures and Tables

**Table 1:** Prediction performance of SNV-preprocessed PLSR models using full-band spectra and selected wavelengths (CARS and UVE).

Input Variables	No. of Wavelengths	LVs	Training R^2^	Training RMSE	Testing R^2^	Testing RMSE
Full-band (SNV)	256	12	0.5980	16.0006	0.5628	15.3791
CARS-selected (SNV)	50	21	0.7296	12.9526	0.6590	14.1218
UVE-selected (SNV)	65	19	0.6868	13.9979	0.5878	15.3732

Notes: SNV, standard normal variate; PLSR, partial least squares regression; CARS, competitive adaptive reweighted sampling; UVE, uninformative variable elimination; LVs, number of latent variables. RMSE, root mean square error.

**Table 2:** Performance comparison of regression models based on CARS-selected wavelengths.

Model	Training RMSE	Training R^2^	Testing RMSE	Testing R^2^
PLSR	12.9526	0.7296	14.1218	0.6590
KNN	0.4499	0.9997	4.8938	0.9341
SVR	11.4546	0.7499	14.4389	0.7426

Notes: PLSR, partial least squares regression; KNN, k-nearest neighbors regression; SVR, support vector regression; CARS, competitive adaptive reweighted sampling; RMSE, root mean square error.

**Table 3:** Performance of RF, SVM, and KNN models for AFB_1_ contamination grading based on predicted values.

Model	Accuracy	Macro-F1	Cohen’s Kappa
RF	88.89%	0.8727	0.8452
SVM	95.24%	0.9440	0.9344
KNN	90.48%	0.8929	0.8690

Notes: Metrics were calculated on the independent test set.

## Data Availability

The original contributions presented in this study are included in the article. Further inquiries can be directed to the corresponding authors.
